# Dealing with information overload: a comprehensive review

**DOI:** 10.3389/fpsyg.2023.1122200

**Published:** 2023-06-21

**Authors:** Miriam Arnold, Mascha Goldschmitt, Thomas Rigotti

**Affiliations:** ^1^Leibniz Institute for Resilience Research, Mainz, Germany; ^2^Work, Organizational and Business Psychology, Johannes Gutenberg-University Mainz, Mainz, Germany

**Keywords:** information overload, information flood, intervention, job design, review—systematic

## Abstract

Information overload is a problem that is being exacerbated by the ongoing digitalization of the world of work and the growing use of information and communication technologies. Therefore, the aim of this systematic literature review is to provide an insight into existing measures for prevention and intervention related to information overload. The methodological approach of the systematic review is based on the PRISMA standards. A keyword search in three interdisciplinary scientific databases and other more practice-oriented databases resulted in the identification of 87 studies, field reports, and conceptual papers that were included in the review. The results show that a considerable number of papers have been published on interventions on the behavioral prevention level. At the level of structural prevention, there are also many proposals on how to design work to reduce information overload. A further distinction can be made between work design approaches at the level of information and communication technology and at the level of teamwork and organizational regulations. Although the identified studies cover a wide range of possible interventions and design approaches to address information overload, the strength of the evidence from these studies is mixed.

## 1. Introduction

With the digitalization of both work and private life, information is available in large quantities in digital form; we live in an “information society” (Karvalics, 2007). It is possible to conveniently and actively access diverse information, and we also passively receive large amounts of information and messages. Despite the different channels of information, information is mostly consumed through screen displays. Bawden and Robinson (2009) refer to this phenomenon as “homogenized diversity.” Currently, the amount of information that is created every two days is roughly equivalent to the amount of information that was created between the beginning of human civilization and the year 2003 (Jackson and Farzaneh, 2012). The amount of information available has thus become excessive, but it is difficult to assess its quality. As a result, information overload has become a widespread problem. Indeed, information overload was cited as one of the most frequent stressors by 22.5% of respondents in a representative German sample (Meyer et al., 2021). The COVID-19 pandemic can be seen as a catalyst for these developments, and it seems likely that the resulting increased use of mobile working patterns, virtual meetings, and collaborative digital software will be permanent (Rigotti et al., 2021).

Empirical evidence shows that information overload is positively related to strain, burnout (Girard and Allison, 2008; Day et al., 2012; Antoni and Ellwart, 2017), and various health complaints (Junghanns and Kersten, 2020), and negatively related to job satisfaction (Hunter and Goebel, 2008). Furthermore, information overload is associated with serious performance losses, especially in connection with disruptions and interruptions (Baethge and Rigotti, 2010; Rigotti, 2016). Finally, studies show that the quality of individuals’ decisions is affected by information overload (Phillips-Wren and Adya, 2020).

Given the relevance of information overload for the health and well-being of employees, as well as their performance outcomes, we provide a systematic review of preventive measures for information overload. Previous reviews on the topic of information overload aimed to identify the factors that influence information overload (e.g., Eppler and Mengis, 2004; Antoni and Ellwart, 2017) or to present the consequences of information overload (e.g., Antoni and Ellwart, 2017). Furthermore, a meta-analysis by Graf and Antoni (2020) focused on information characteristics as antecedents of information overload. Although some review articles have examined the design or intervention options to counteract information overload, these are usually specific to one professional group (mainly medicine, e.g., Khairat et al., 2018).

Firstly, the aim of this review is to systematically describe the tools and interventions that can be used to manage information overload. The various recommendations and interventions are clustered according to the levels of the causes (Eppler and Mengis, 2004) or sources (Graf and Antoni, 2020) of information overload. This clustering makes it possible to determine whether the recommendations address the amount of incoming information or, conversely, the handling of the incoming information. In addition, this study assesses the extent to which preventive measures are proposed in terms of behavioral versus design solutions.

Secondly, we aim to assess the current state of knowledge on design recommendations for information overload. We will also examine whether the guidelines and recommendations are concrete or still relatively vague and, thus identify the areas in which knowledge deficits remain.

## 2. Theoretical classification and definitions

Several papers have already addressed the theoretical grouping of the factors that contribute to information overload and the consequences of information overload (Eppler and Mengis, 2004; Graf and Antoni, 2020). These models thus provide a theoretical framework for the present review. In addition, the cognitive load theory (Atkinson and Shiffrin, 1968) and the media richness theory (Daft and Lengel, 1986) are often referred to in the literature on information overload. Therefore, we will briefly describe these theories.

In their framework model of the concept of information overload, Eppler and Mengis (2004) suggest that there are several interrelated causes of information overload: the characteristics of the person receiving the information, the characteristics of the information, tasks and processes, organizational processes, and information technology. The resulting consequences of information overload require the use of countermeasures, which, in turn, affect the causes of information overload. This process is circular, and all the aspects are interdependent (Eppler and Mengis, 2004).

Cognitive load theory suggests that the human working memory is limited to approximately seven ± two units of information (Atkinson and Shiffrin, 1968). Accordingly, information overload occurs when the amount of information exceeds the working memory of the person receiving it (Graf and Antoni, 2020). Cognitive load theory identifies three different categories of cognitive load: extraneous, intrinsic, and germane cognitive load. Extraneous cognitive load is influenced by the design of the information (Sweller, 2005). Intrinsic cognitive load results from the content of the information, such as its complexity. Finally, germane cognitive load is the favorable, learning-enhancing cognitive load that results from focused engagement with the information. Ideally, this final form of cognitive load leads to the construction of schemata and mental models (Sweller, 2005).

Media richness theory also provides a theoretical framework for studying information overload. One of the goals of using information and communication technology (ICT) is to reduce the ambiguity of information (Daft and Lengel, 1986). Therefore, information richness, which refers to the extent to which information can change understanding in a given time interval (Daft and Lengel, 1986), should be considered in relation to information overload. In addition to the information itself, the various possible communication channels may also differ in their richness. Face-to-face conversations are thought to be the richest form of communication, while letters or e-mails are less rich (Daft and Lengel, 1986; Kauffeld et al., 2016). Cognitive load theory provides a precise definition of information overload, while the media richness theory better supports the development of design interventions. Against the background of these relevant theories, the terms used in this study can be defined.

Information overload is a topic that is relevant to many disciplines, including medicine, social sciences, marketing, computer science, education, and psychology (Edmunds and Morris, 2000). This means that there is no single, universally accepted definition. In everyday language, information overload is often equated with receiving too much information (Eppler and Mengis, 2004). According to Klausegger et al. (2007), a consistent feature of the various scientific definitions of information overload is that the amount of information is initially related to better performance or better decisions but that, above a certain amount of information, the effect changes, and the amount of information leads to worse outcomes (an inverted U-shaped relationship; Eppler and Mengis, 2004; Klausegger et al., 2007). More specifically, Klapp (1986) defined information overload as an excessive amount of information that the receiver can no longer process efficiently without distraction, stress, increased errors, or other costs that reduce the efficient use of the information. Similarly, Eppler and Mengis (2004) argued that information overload occurs when the amount of information exceeds the processing capacity of the recipient.

As mentioned above, information overload is closely related to the use of ICT and, therefore, also to the concept of technostress. Technostress can be defined as the “stress experienced by individuals due to the use of ICTs” (Ragu-Nathan et al., 2008, p. 418). Information overload and constant availability are the two main stressors caused by the use of ICTs (La Torre et al., 2019). Therefore, information overload can be seen as a feature of technostress. Although this review focuses on information overload, it also includes primary studies that refer to both technostress and information overload. Five techno-stressors are commonly discussed in the technostress literature. These are techno-overload (leading employees to work longer and faster), techno-invasion (constant availability, including in leisure time), techno-complexity (complexity of the digital tools leading to feelings of inadequacy of computer skills), techno-insecurity (threat to the security of one’s job), and techno-uncertainty (constant upgrades of hardware and software) (La Torre et al., 2019). Factors with the potential to buffer the detrimental effects of techno-stressors on employee well-being are discussed under the label of techno-inhibitors (Ragu-Nathan et al., 2008; Estrada-Muñoz et al., 2022). Examples of techno-inhibitors are literacy, participation, or innovation support (Jena, 2015; Estrada-Muñoz et al., 2022).

## 3. Methods

### 3.1. Search strategy

Following the recommendation by Methley et al. (2014) we used the PICO tool in order to define our keywords in both English and German (see Table 1). As the discussion on information overload comes from several fields, many synonyms for information overload are used. To cover these, we included a large number of synonyms as outcomes in our search string, linked with an OR operator. As we are interested in methods and interventions to reduce information overload, a further part of the search string refers to aspects that could be used as countermeasures against information overload. In order to also cover a wide range of potential countermeasures, we included ‘software’ as a keyword beneath the more psychological aspects of intervention/training/workshop or work/task design. In addition, to address individual strategies, we included the keyword ‘coping strategies’. As described, the focus of this review is on information overload in the workplace, so a third set of keywords defining the population was added to ensure the relevance of the search results to the workplace. In order not to narrow down the results further than necessary, keywords in the comparison category were omitted.

**Table 1:** Search string used in scientific and applied databases based on the PICO concept.

	Search string used for English language databases	Search string used for German language databases
**P**opulation	(work OR occupation OR job OR employment)	(Arbeit OR Beruf OR Job OR Beschäftigung OR Anstellung OR Erwerbstätigkeit OR Tätigkeit OR Zusammenarbeit)
	AND	AND
**I**ntervention	(intervention OR training OR workshop OR software OR countermeasure OR remedy OR solution OR “work design” OR “work redesign” OR “task design” OR “task redesign” OR “organization design” OR “job design” OR “coping strategies”)	(Intervention OR Interventionsmöglichkeiten OR Training OR Workshop OR Software OR Gegenmaßnahme OR Lösung OR Abhilfe OR Arbeitsdesign OR Arbeitsgestaltung OR Arbeitsumgestaltung OR Aufgabendesign OR Aufgabengestaltung OR Aufgabenumgestaltung OR Organisationsgestaltung OR Strategien OR Mechanismen)
	AND	AND
**C**omparison	n/a	n/a
**O**utcome	(“information overload” OR “flood of information” OR “information surplus” OR “information avalanche” OR “data trash” OR “data smog” OR “information input overload” OR “information burden” OR “data explosion” OR “technostress”)	(Informationsüberlastung OR Informationsflut OR Informationsüberladung OR Informationsüberflutung OR Datenexplosion OR Datenmüll OR Technikstress)

In the second step, we defined the databases to be searched. We searched the scientific databases Web of Science, Ebscohost, Medline, PsycInfo, and PsycArticles using complete search strings with Boolean operators. With the aim of including the applied literature, we also searched other practical databases, including PSYNDEX Interventions, Rehadat, Arbeitssicherheit.de, the BAuA (Federal Institute for Occupational Safety and Health) publications, and publications of the Verwaltungs-Berufsgenossenschaft (VBG). As most of these platforms do not allow searches using Boolean operators, all the synonyms for information overload were used individually as keywords.

**Figure 1:**
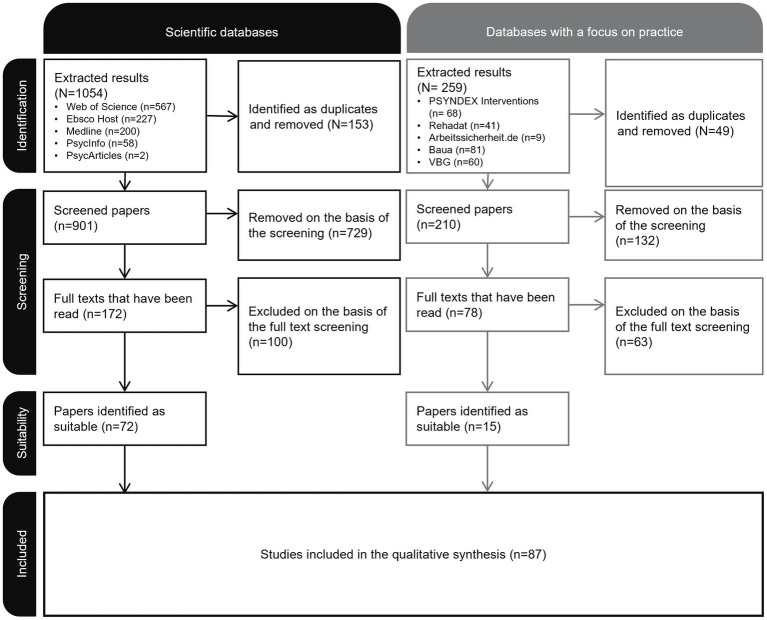
PRISMA flowchart.

In the subsequent review process, we followed the PRISMA standards (Moher et al., 2009) and, accordingly, recorded how many results were obtained from the literature searches conducted in this way (identification, see Figure 1). Following the literature search, all duplicates were removed, and two independent raters classified the search results as relevant or irrelevant to the research question on the basis of titles and abstracts (screening). The screening process itself was blinded. If the two raters disagreed about certain identified documents, we made a joint decision about inclusion or exclusion. We then read the full texts of the documents that were deemed relevant which led to further exclusions if they did not fit with the research question (suitability).

In the final step, we coded and categorized the content of the eligible documents. We recorded the level of the intervention (person, information, tasks and processes, organizational processes, information technology), a precise description of the intervention, the type of article, the research question, the design of the study, the sample, the results of the study, and the implications in terms of recommendations for action and the person who should implement them. Excerpts of the included sources are provided in Tables 2–6).

**Table 2:** Results of the literature search at the information level.

Citation	Method	Context	Design & Sample	Recommendation for action	Initiation
Quality and presentation of information					
Beasley et al. (2011)	Concept	Medicine	-	Describes the problem of information chaos in the medical field	-
Khairat et al. (2018)	Review	Medicine	*N* = 17 individual studies	The use of dashboards to visualize patient data is recommended. They reduce data collection time, data collection difficulties, cognitive load, task completion time, and the number of errors. At the same time, they improve situational awareness, safety compliance, usability, and navigation.	O
Waller et al. (2019)	Review	Medicine (intensive care)	*N* = 22 individual studies	Four different types of information displays are being investigated in the field of intensive care medicine: Integrated displays Dashboards integrating several patients’ information Monitoring of physiological and laboratory values Expert systems with decision support The review concludes that only 12 of the 22 studies measured a significant improvement in one of the primary outcomes. Therefore, no clear recommendation for use can be made.	O
Patapovas et al. (2013)	Quant & Qual	Medicine, Germany	*N* = 9 doctors and use of the tool for 752 patients	Bundled presentation of clinical information in a decision support system with four aspects: Presentation of the current medication Info button for drugs and their most common problems Medicheck = contraindications, interactions with other drugs More in-depth information on pharmaceuticals available with one click Most test users used the tool daily and rated it positively (usefulness, importance, ease of use).	O
Ahmed et al. (2011)	Quant	Medicine	*N* = 20 intensive care physicians processing eight patient data each	Use of electronic patient records. The results of the study show that reducing, filtering, and better categorizing of patient data can improve performance and reduce information overload. Time pressure was considered an outcome variable in the study.	O
Tan et al. (2013)	Quant	Medicine, Singapore	*N* = 196 nurses in five wards	A dashboard of relevant patient information bundles urgent orders, abnormal lab or radiology results, and infection alerts. Additional functionality is provided for the discharge process and preparation for radiological exams. The testers were very satisfied with the tool and particularly appreciate the time savings and efficiency and the increased patient safety provided by the tool.	O
Wu et al. (2016)	Quant	Production, China	*N* = 38 operators of an LED manufacturer	The presentation of information at the human-machine interface should be of low complexity. This reduces the cognitive load on the user.	O/EX
Ries and Deml (2019)	Hand	Control rooms, Germany	−	The work shows how information in the form of video recordings can be optimally presented to reduce the strain of control room staff.	O
Wnuk et al. (2016)	Qual	Software development, Sweden	*N* = 11 testers	The FSC+ software enables the visualization of process steps in larger projects, thus facilitating the analysis of project progress and can thus contribute to decision-making. The survey of sample users showed that its use is recommended for people who need to keep track of many project steps.	O/EX
Roy et al. (2017)	Review	Management bodies in companies	design science research (DSR)	Solutions at the knowledge level: summaries, expert comments, use of indicators, prioritization, distribution of information over time, visualization of information. Solutions based on the use of digital media: search engines, blogs, dashboards, news, and updates, taking notes and using virtual bookmarks, using alerts.	EM/L/EX
Dzokoto et al. (2013)	Concept	-	Proposal based on a literature review	“SMART Push System”: actively suggests relevant documents/content to decision-makers, taking into account all available information/contextual knowledge, without them having to actively request the information.	EX
Riener and Ferscha (2008)	Qual	-	*N* = 10 persons	Wearing a vibrotactile belt, that can provide additional tactile information, here applied to distance perception. However, no findings are reported on how this contributes to reducing information overload.	EX
Quantity of information					
Shah et al. (2019)	Quant	Medicine, USA	*N* = 8,411 employees from 148 facilities of the Department of Veterans Affairs	Introduction of a new organizational policy for sending information and messages, combined with training of employees on the setting options. This reduces the number of messages received.	O
Pickering et al. (2013)	Quant and Qual	Medicine (intensive care unit), USA	*N* = 925 questionnaires answered by ICU staff on new admissions	The study shows that out of a total of 51 concepts, an average of 11 are used for each patient. Four concepts are used particularly often. From this observation it can be concluded that certain information should be presented in a prioritized way. Access to other information should not be prevented, but the most frequently used categories of information should be presented in a clear and concise manner.	O/L
Arditi et al. (2017)	Review	Medicine	*N* = 34 individual studies	Use of computer-generated reminders in paper form.	L/O

Quant, quantitative study; Qual, qualitative study; review, review paper (systematic or unsystematic); Meta, meta-analysis; Concept, concept paper or opinion; Hand, handout, rule or information; EM, employees; L, leaders; T, teams; O, organization; EX, external support (e.g., IT staff or employees of a consultancy).

**Table 3:** Results of the literature search on the level of the person.

Citation	Method	Context	Design & Sample	Recommendation for action	Initiation
Dealing with information overload					
Mustapar et al. (2016)	Review	-	*Thematic analysis* following Miles and Huberman (data collection, reduction, presentation and drawing)	Information overload should be identified as early as possible in order to take preventive action.	EM/L/O/EX
Landale (2007)	Concept	-	-	Information overload should be addressed proactively at the individual level. A 3-step approach is presented: - Step 1: Receiving the document, assessment needed - Step 2: Initial understanding of the document - Step 3: New knowledge is adapted	EM
Warbington (2000)	Concept	-	-	It is necessary to clarify the goal one is pursuing. Based on this, the importance of different information should be assessed.	EM
Farhoomand and Drury (2002)	Qual	Australia, Hong Kong, UK, USA	Questionnaire with five questions (*N* = 124 managers)	Filtering information (suppressing, deleting, and selecting irrelevant information) helps to reduce information overload. In addition, work can be delegated, especially for screening or filtering tasks. It is also recommended to prioritize information.	EM
Kluge et al. (2020)	Concept	Germany	-	At the individual level, it is recommended to filter information and to delegate tasks from humans to digital agents.	EM
Stich et al. (2018)	Review	-	-	At the individual level, interventions such as e-mail training, virtual courtesy, respect, and engagement workshops are recommended. In addition, individuals should develop mindfulness toward themselves (e.g., measuring and reporting levels of technostress or attitudes toward ICT through surveys) and toward colleagues. Managers have a special responsibility here, but employees should ask their colleagues which communication channels they prefer.	EM/L
Training					
Drössler et al. (2018)	Review	-	34 studies (thereof 18 Qual)	Extraction of design recommendations at various levels, including company-specific information policy, training for efficient use of e-mail programs, active information search vs. push messages, training for media and information literacy, avoidance of multiple channels, and disruption-free times.	EM/L/O
Stadin et al. (2020)	Qual	Sweden, health sector	Interview with Critical Incident Technique (*N* = 20)	Promoting a good e-mail culture, training individual strategies and competencies for using e-mail and software in general.	EM/L/O
Pfaffinger et al. (2020)	Qual	International; German, English, Spanish	Semi-structured interviews (*N* = 26 employees)	It is recommended to participate and proactively request training, to trust in the adaptation process, to consciously set limits, and to organize the working day efficiently.	EM/L
Antoni and Ellwart (2017)	Review	-	Review along the model of Eppler and Mengis (2004)	Training measures for competencies in modern information and communication technologies are recommended. People should be made aware of the adverse effects of e-mail interruptions and be taught concrete techniques for dealing with e-mails efficiently. For example, it may be helpful to establish fixed times for dealing with e-mail.	EM/L
Nagarajah (2016)	Quant	-	Questionnaire (*N* = 212)	Accompany the introduction of new technologies with appropriate training offers and support.	L/O
Lehman and Miller (2020)	Review	-	“framed humanist review”	Depending on the type of information search, the appropriate digital competencies should be trained, and recommendations for action should be followed.	EM/L
Bundesanstalt für Arbeitsschutz und Arbeitsmedizin (BAuA) (2006)	Hand	Germany	-	Practice information culture in the company (e.g., improve quality of e-mails, reduce quantity/scope, internal rules); provide specific training for employees (e.g., media competence, time management, e-mail etiquette); existing training from the initiative “New Quality of Work.”	EM/L/O
Benselin and Ragsdell (2016)	Quant & Qual	-	Interviews (*N* = 5), Diaries (*N* = 4), questionnaire (*N* = 46)	Information literacy training is recommended.	EM/L
Cheuk (2008)	Qual	Consulting firm	a consulting firm	Information literacy is a human resource development issue; 4 dimensions are proposed: skillful use of information at a strategic level, organization and control of information, knowledge of access and tools, linking search and use of information.	O
Yener et al. (2020)	Quant	Public service	Cross-section (*N* = 138)	Promoting technology self-efficacy and time management can counteract the adverse effects of technostress on burnout and performance.	EM/L
Mahapatra and Pati (2018)	Quant	India	Questionnaire (*N* = 163)	Reduce techno-uncertainty and techno-complexity, e.g., through regular training or seminars.	EM/L
Le Roux and Botha (2021)	Quant	South Africa, Economy	Cross-section (*N* = 106 managers)	Support in the reduction of techno-complexity for older employees, e.g., through training courses.	L
Zhao et al. (2020)	Quant	-	*N* = 513	Starting points for interventions: cognitive assessment of technostress and problem-focused coping or emotion-focused coping (venting).	EM/L
Becker et al. (2021)	Quant	Germany	Cross-section, questionnaire, *N* = 3,363 knowledge workers	Coping strategies reduce the negative correlation between technostress and performance, so different coping strategies should be trained. Active-functional strategies are to be preferred. The negative effects of dysfunctional coping strategies should be explained.	EM/L
Yin et al. (2018)	Quant	Internet industry	Cross-section, *N* = 178	*Information processing timeliness* (=degree of perceived support in timely information processing by mobile information and communication technologies) is not recommended as a coping option.	-
Moser et al. (2002)	Qual & Quant	mainly in the service sector	Pre-study (*N* = 12 interviews) Main study (*N* = 159 one-time questionnaires), study on e-mail programs (review), study on the use of communication aids (*N* = 488, one-time questionnaire), study on organizational strategies (*N* = 24 interviews)	“Resources for dealing with information overload”: adequate technical equipment, information culture in the company, social support, media competence, and work methodology. From the qualitative study, several lived measures and strategies emerge, including acceptance and optimization, rejection and avoidance, selection strategies. The authors develop a training concept based on the results of the study. The training includes the aspects of know-how, efficient handling of information overload, time management methods, training on e-mail programs, addressing the 11 factors of information overload as a stressor, and workplace-related individual coaching.	EM/L/O
Soucek and Moser (2010)	Quant	–	Intervention study without control group with pre-test and double post-test design, *N* = 90 employees	Training (knowledge, use of e-mail features, media use problems, work disruptions, and e-mail stress) to improve e-mail communication has been shown to be effective and is therefore recommended.	EM/L
Moser and Soucek (2005)	Concept	–	–	A needs analysis is required before training can be offered within a company. The importance of practicing what has been learned is emphasized. It is also recommended that people who work together in the company attend the e-mail communication training together so that an e-mail culture can develop.	L/O
De Bruin et al. (2020)	Quant	Netherlands, Economy	Wait-control group; pre-post-follow-up design (*N* = 150)	Positive effects of mindfulness training on stress (measured by the Perceived Stress Scale, PSS; Cohen et al. 1983), work engagement, and “Checklist Individual Strength”; but not directly related to information overload.	EM/L
Camargo (2008)	Qual	High-Technology	Interviews (*N* = 17 “High Technology” Workers)	Creative solutions for the further training of employees are recommended, such as instructional videos on how to use e-mail efficiently. In addition, several steps to reduce e-mail stress are presented: Setting realistic goals regarding the number of tasks, developing rules and filters for e-mail use, using alternative communication channels, and separating work and private life.	EM/L/O
Dealing with E-mails and the Internet					
Ramsay and Renaud (2012)	Qual	Different Industries	Semi-structured interviews in individual setting (*N* = 18); focus group with 3 experts to develop interview guide	Communicate the expectation of responding quickly. No expectation to read e-mails outside of work hours and accept different handling of the frequency of reading e-mails (against compulsive checking). “Ignore and delete”. Only forward of CC someone when necessary. Avoid “buck-passing”: do not pass the task to someone else by forwarding an e-mail. Avoid “broadcasting”: send to a large distribution list only when necessary; otherwise, send individual requests. BCC (“blind copying”) should remain allowed, as there are usually good reasons for doing so. Send one well-thought-out e-mail, not multiple or periodic follow-ups.	EM
Soucek (2009)	Review	-	Literature review	Structure and manage incoming mail Timing of e-mail communication Media-appropriate use of e-mail correspondence Rules for e-mail communication	EM
Bundesanstalt für Arbeitsschutz und Arbeitsmedizin (BAuA) (2011)	Hand	Germany	-	Create pauses and downtime (e.g., turn off your e-mail inbox to avoid interruptions). Concrete tips to avoid information overload concerning e-mails, including precise formulation of concerns based on the subject line, communicating the further need for action, standard templates, avoiding CC and forwarding, and keeping the amount of information as small as possible. Concrete tips against information overload regarding the Internet, including precise keywords, checking for up-to-dateness, filing system, and direct communication to be considered	EM
Rack et al. (2011)	Quant	Pharmacy, Software	Interviews (*N* = 40)	Optimization options against e-mail information overload: On the recipient’s side: automatic filtering of incoming mail, individual strategies, e.g., cross-reading On the sender’s side: question the necessity of the e-mail, send only to relevant recipients, and choose precise wording.	EM
Active design of the workplace					
Gaudioso et al. (2017)	Quant	USA	Cross-section, questionnaire, *N* = 242 employees (use of IT at work)	Maladaptive coping strategies should be eliminated, and adaptive ones subsequently built upon. Raise awareness and remove barriers (e.g., through training, job design, reward systems, peer pressure, and technical support). Technostress can be reduced, for example, by not working on e-mails outside working hours.	EM/L

Sources in italics were mentioned in multiple of the running text’s sub-chapters at the individual level. However, these are only listed once in the tabular overview. Quant, quantitative study; Qual, qualitative study; review, review paper (systematic or unsystematic); Meta, meta-analysis; Concept, concept paper or opinion; Hand, handout, rule or information; EM, employees; L, leaders; T, teams; O, organization; EX, external support (e.g., IT staff or employees of a consultancy).

**Table 4:** Results of the literature search on the level of tasks and processes.

Citation	Method	Context	Design & Sample	Recommendation for action	Initiation
Piecha and Hacker (2020)/Piecha (2021)	Qual & Quant	Germany	Multimethod interviews (*N* = 73), cross-sectional survey (*N* = 320), diary study (*N* = 93); 19 design workshops	The comprehensive recommendations for action derived from these studies are presented in Figure 3. Time pressure is identified and examined as an influencing factor for information overload.	EM/L/O
Stadin et al. (2020)	Qual	Sweden, healthcare managers	Critical Incidents, *N* = 20 healthcare managers	Promote a good e-mail culture, train individual strategies and competencies for dealing with e-mails and software in general and provide good IT support.	O
Stich et al. (2018)	Review	-	-	Establish clear policies on ICT use (e.g., block e-mail traffic outside working hours; switch from e-mail to an internal social network, establish policies against cyberbullying or on ICT usage in general). Individual interventions (e.g., e-mail training, workshops on virtual courtesy, respect, and engagement). Creating mindfulness towards oneself (e.g., measuring and providing feedback on levels of technostress or attitudes towards ICTs through questionnaires). Create mindfulness towards colleagues (special responsibility of managers, but also ask colleagues which communication channels they prefer).	O
Bordi et al. (2017)	Qual	Finland, office work	Group discussions (*N* = 36)	Improve information ergonomics: establish standard rules (etiquette), trouble-free times, training in the use of tools, prioritization.	EM/T/O
Kluge et al. (2020)	Concept	Germany	-	Individual/team level: (a) filter information, (b) delegate tasks from people to “digital agents”. Organizational level: (a) use of management information systems, (b) use of interactive support systems, (c) “organizational unlearning” = forgetting information that is no longer up to date; be prepared for the consequences of these measures (emerging challenges, e.g., acceptance of technology).	T/O
Okolo et al. (2018)	Quant	Nigeria, Banks	Cross-sectional, questionnaire; *N* = 319 front desk staff in the Nigerian banking sector	Technostress (overload, complexity, and invasion) emerged as a challenge demand. Activity resources showed a positive indirect relationship with work engagement mediated by technostress. Time and performance pressure are described as part of the problem of technostress.	-
Oldroyd and Morris (2012)	Concept	USA, “Star employees” (High performers)	–	To reduce information overload for “star employees” with an extensive social networks, the following recommendations are offered, among others: Person: Training in the ability to distinguish between useful and superfluous information; creating schemas to support long-term memory; motivational support; scope for action. Tasks & processes: support employees for star employees (gatekeepers); reduce the width of the star network; increase the density of the star network; distribute standardized information to the entire network instead of individualized information. Organizational processes: Clarity about where to find information, cost of sharing information; using information systems to contextualize information and make it more useful to employees; creating dedicated knowledge-sharing positions for star employees; encouraging relationships with other star employees.	L/O
Coorporation in teams					
Ellwart et al. (2015); see also Happ et al. (2015).	Quant	Germany, office work	Experimental design, measurement Team Mental Model, information overload, and number of e-mails *N* = 363 students	STROTA (structured online team adaptation): An online intervention for structured team reflection related to information overload. The intervention consists of three steps: individual attentiveness to the situation, attentiveness to the situation in the team and plan formulation.	T
Norri-Sederholm et al. (2015)	Qual & Quant	Finland, Emergency Medicine	Interviews with *N* = 10 emergency physicians with management responsibility	The importance of situational awareness is emphasized.	L
Paul and Nazareth (2010)	Quant	USA	Experimental *N* = 225 Bachelor students who solved decision-making tasks in 45 groups of 5	The advantages of the schematic summary of information seem to emerge only with moderate amounts of information (complexity).	T/EX
Wang et al. (2007)	Quant	Germany	*N* = 5 testers in a working group of developers	The study provides only weak evidence that automatic adjustment of notification rules can be helpful in virtual collaboration.	EX/O
Bergström et al. (2010)	Qual	Sweden, Shipping	Simulation of an escalating emergency situation on a passenger ship, 15 lay and expert teams (maritime and aviation)	Central to effective team action in escalating hazardous situations appears to be a reduction in the amount of information available for decision-making.	T
Ferreira et al. (2011)	Quant	Portugal	Experimental – synchronous brainstorming task in a group, *N* = 55, mainly students	Selective or delayed information about the task status of team members (during individual activity) increased team performance.	T
Drössler et al. (2018)	Review	-	34 studies (thereof 18 Qual)	Extraction of design recommendations on various levels, e.g., internal company regulations on media use (e.g., turning off notification signals during meetings), avoidance of duplicate communication through multiple channels.	EM/L/O
Leadership					
Becker (2009)	Qual	USA, health sector	Interviews with *N* = 12 managers	Leaders’ recommendations: Limit organizational priorities, clarify on project responsibilities and decision-making, fewer and better-structured meetings, “skill set match,” and technology policies.	L
Spagnoli et al. (2020)	Quant	Italy, administrative staff	Cross-sectional questionnaire, *N* = 339 employees of a university administration	Avoid authoritarian leadership, especially where there is a high proportion of home-based work, as this increases feelings of technostress.	L
Mill (2010)	Concept	UK	Own experience (advising companies and managers on dealing with work-related stressors in general)	Effective communication, employee development training, coaching, mentoring, and good leadership/inspiration have a positive impact on work culture and can lead to improved health and well-being.	L

Sources in italics were mentioned in multiple of the running text’s sub-chapters at the task and process level. However, these are only listed once in the tabular overview. Quant, quantitative study, Qual, qualitative study; review, review paper (systematic or unsystematic); Meta, meta-analysis; Concept, concept paper or opinion; Hand, handout, rule or information; EM, employees; L, leaders; T, teams; O, organization; EX, external support (e.g., IT staff or employees of a consultancy).

**Table 5:** Results of the literature search on the level of design of organizational processes.

Citation	Method	Context	Design & Sample	Recommendation for action	Initiation
Manwani et al. (2001)	Review	-	-	Introduction of behavioral and action guidelines can counteract information overload.	O/EX
Remund and Aikat (2012)	Review	-	-	Strategic use of intra-corporate communication can reduce the risk of information overload.	O/EX
Sumecki et al. (2011)	Quant	England	Cross-sectional, questionnaire, *N* = 710 employees of a company in the technology sector	Establish clear company-internal policies on e-mail use to avoid differences among employees in the assessment of e-mails as “business-critical” and to prevent e-mail information overload.	O/EX
Kluge and Gronau (2018)	Review	-	-	Intentional forgetting should be used in change processes to counteract information overload, e.g., by removing various sensory, contextual, or process-related cues.	O/EX
Day et al. (2012)	Quant	-	*N* = 258 employees from various companies and sectors	Providing up-to-date software and timely software updates, as well as personal support from IT staff, can mitigate the negative effects of information overload from ICT use.	O/EX
Farhoomand and Drury (2002)	Qual & Quant	Australia, Hong Kong, UK, USA	*N* = 124 managers	Recognize the dimensions of information overload (extent, irrelevant information, time constraints, variety of information channels) to provide targeted support to affected employees; provide better tools and techniques to better process information from internal and external sources; organizational structure: flatter hierarchies combined with smart IT.	L/O
Florkowski (2019)	Quant	USA	*N* = 169 North American companies represented by HR executives	Manage expectations about the use of technology in HRT; adapt the departmental climate (flexibility, risk-taking, evidence-based experimentation, support for innovation).	L/O
Gaudioso et al. (2017)	Quant	USA	Cross-sectional, questionnaire, *N* = 242 employees (use of IT at work)	Coping strategies: eliminate maladaptive coping strategies and then build adaptive ones, increase awareness & reduce barriers (e.g., through training, job design, reward systems, peer pressure, and technical support). Reduce technostress (e.g., no e-mail outside work hours, improve organizational culture).	L/O
Pfaffinger et al. (2020)	Qual	International; German, English, Spanish	Semi-structured interviews with *N* = 26 employees	Recommendations at the level of organizational processes: invest in IT security, communicate, structure, and support the introduction of new technologies well, develop new work concepts, clarify expectations regarding flexibility of time and place, ensure compliance with work rules, provide ergonomics also in the home office, provide the latest technologies and the corresponding technical support, provide helpdesk and management training, provide training for individual employees. Recommendations at the level of society: Ensure participation, public training, or helpdesks, ensure high-speed Internet for mobile phones, prohibit or regulate surveillance, protect data rights, punish violations, offer social services/security in case of job loss, ensure humanity of new forms of work, a legal framework for separation of work and private life, offer functioning infrastructure for companies.	L/O
Soucek (2017)	Quant	Germany	*N* = 264 employees	Agreements on e-mail communication are considered valuable, especially when made at the departmental level.	L/O
Stich et al. (2018)	Review	-	-	Establish clear policies on ICT use (e.g., block e-mail traffic outside working hours; switch from e-mail to an internal social network, establish policies against cyberbullying or on ICT use in general).	O

Quant, quantitative study; Qual, qualitative study; review, review paper (systematic or unsystematic); Meta, meta-analysis; Concept, concept paper or opinion; Hand, handout, rule or information; EM, employees; L, leaders; T, teams; O, organization; EX, external support (e.g., IT staff or employees of a consultancy).

**Table 6:** Results of the literature search at the level of information technology.

Citation	Method	Context	Design & Sample	Recommendation for action	Initiation
Graf and Antoni (2020)	Meta	-	Quantitative analysis with *N* = 24 studies	The type of technology may influence the relationship between information characteristics and information overload. Instead of quantity of information, more attention should be paid to quality in order to reduce information overload; the choice of the appropriate technology may already be relevant for the transmission of information (Media Richness Theory).	O/EX
Use of information and communication technology					
Ayyagari et al. (2011)	Quant	USA, working people in all sectors	Cross-sectional survey, *N* = 661 employees via online panel	The study shows that dynamic changes in ICT are particularly associated with employee stress via work stressors. Accordingly, dynamic changes in ICT should be considered as a stress factor.	L/O
Drössler et al. (2018)	Review	–	34 studies (thereof 18 Qual)	Extract design recommendations at various levels, including thoughtful consideration of the introduction of new ICT and care in the selection of ICT for appropriate information provision.	L/O
Kauffeld et al. (2016)	Concept	–	–	According to the Media Richness Theory, it can be recommended that depending on the content of the information, the appropriate rich form of communication should be chosen. For example, when getting to know new colleagues, face-to-face communication may be preferred as the richest form, whereas an appointment can also be confirmed by e-mail. The information content of the communication medium and the content of the information should therefore match as closely as possible.	EM/L
Oehme et al. (2019)	Qual & Quant	Work in control rooms, Germany		The use of ICT in this occupational field allows the digitization of processes and increases the freedom of movement. The exchange of information via ICT seems to be more effective and efficient than before the use of ICT. The exchange of information via ICT goes beyond communication via telephone/radio and seems to make work easier. Participants did not report information overload through the use of ICT. Time pressure is considered as an outcome.	L/O
Warren (2014)	Review (not systematic)	–	–	A unified file system for all personal and shared information. Use of tagging in addition to traditional hierarchical folder structures. Control of a user’s contextual information to support information retrieval and reduce overload when switching between contexts. Use semantic technology (e.g., machine learning or NLP to facilitate information retrieval and sharing between applications).	L/O
Jackson and Smith (2012)	Qual & Quant	Software development company, UK	Focus group discussion, *N* = 5	The study shows that the traditional hierarchical system leads to more irrelevant or no information when retrieving information, which is reduced when tagging is used. Companies should therefore check whether the operating system and software allow the use of tags and make greater use of them.	O/T
Demirsoy and Petersen (2018)	Qual	Development department of a company, Sweden	Interviews with *N* = 8 persons	Implementation of a semantic knowledge management system consisting of four components: Text processing Ontology and knowledge base Semantic annotation and ontology filling Semantic search. The system was positively evaluated by those who tested it.	O/T
Celi et al. (2014)	Review (not systematic)	Medicine	-	The authors derive ten suggestions for the design of data systems in medicine from their literature review: (1) Automatic integration of data (2) Collection and integration of the newly available and historical data (3) Step by step display of diagnostics, therapeutic, prognostic, and other documentation suggestions are displayed step by step based on the combination of various data. (4) Machine learning (5) Individual customization (6) Data aggregation for research (7) Reports with “best practice” recommendations (8) The system should be changeable (9) Scalable to integrate new data (10) Test a prototype	EX
Martignene et al. (2020)	Concept	Medicine	Evaluation of a prototype by two experts based on 24 clinical cases	Development and evaluation of the “Heimdall” prototype for the development of simple visualization methods for medical data in the context of care and research. The evaluation showed that complex and heterogeneous data can be displayed clearly, filters facilitate the work, and user settings are possible. The program is open source and can be used via R.	O/EX
Fellmann et al. (2019)	Quant	Employees	Questionnaire, one survey time point, *N* = 103	Implementation of stress-sensitive IT systems makes sense; consider user needs, such as: Acceptance of measurement methods (questionnaire self-assessment most popular, use of calendar/document/communication data accepted, neurophysiological measurements least accepted) Accepted purposes of data collection (individual use and improvement of work organization) Feedback (depends on the work environment, privacy concerns, the system should recognize current work environment, communicate stress status directly with countermeasures, if necessary, only moderate interest in predicting future stress status) Preferred mode of interaction (semi-automated systems that inform users about workload and stress and offer countermeasures only after prompting)	O/EX
Filter and decision support systems					
Ulfert et al. (2022)	Quant	Students and employees	2 Vignette studies comparing different autonomous decision support systems Study 1 with *N* = 244 persons, Study 2 with *N* = 500 persons	The application of a decision support system to e-mail inboxes is recommended, especially more experienced employees may benefit from such a system; no positive effects could be shown for the application to customer service, including telephone service; the participation of potential users in the introduction of such a system is recommended to minimize the possible adverse effects, parallel training of technological and work-related skills may increase positive effects to reduce uncertainties in the use of intelligent systems	EX
Garnsworthy et al. (2004)	Qual	Control room in the metro surveillance	Testing of a prototype for a decision support system	The system can monitor, predict, and suggest improvements. The use of the system appears to reduce information overload and increase the effectiveness in the control room. This technique is applicable to several other control rooms.	EX
Mack et al. (2009)	Review	Medicine (Paediatric Intensive Care Unit)	Included are English-language studies that address decision support systems	Decision support systems can improve clinical work in pediatric intensive care units. However, potential adverse effects of implementing new technologies in complex healthcare settings must be considered.	O/EX
Algorithms for summarizing or extracting information					
Sappelli et al. (2016)	Concept	Office work	Testing of an automated system of categorization of e-mail content based on two sample datasets of e-mail traffic	The proposed algorithm can categorize the content of an e-mail according to the sender’s intent of the sender, the response expectation, the sender’s authority and the number of tasks included. No test usage.	O/EX
Zhang and Xiao (2018)	Concept	Working with texts	Testing of an algorithm, which summarizes sections of a text in a key sentence each, based on five sample data sets	The newly proposed algorithm cosnistently delivers the best results compared to other algorithms. No test usage.	O/EX
Automating monitoring tasks					
Tung et al. (2011)	Concept	Surveillance through cameras in public spaces	Testing of an algorithm based on three sample data sets	Automate the analysis of video images to reduce the burden on staff who must monitor multiple camera angles simultaneously. The goal is to automatically point out people whose movement patterns in public spaces deviate from predictable movement patterns. Although the algorithm delivers good results, the false positive rate is relatively high (1.8–22%). No test use.	O/EX
Hassan et al. (2019)	Quant	IT security	Testing with sample data set and application with *N* = 191 test beneficiaries over five days	A program (NoDoze) that ranks incoming security warnings from an “intrusion detection system” according to urgency and successfully ranks potential false positives very low. It reduces the analysis time for a (simulated) attack.	O/EX
Baumgartner et al. (2014)	Concept	Control room in the Traffic monitoring	-	Description of the BeAware software for increasing situational awareness at traffic monitoring control centers. No testing of the software.	O/EX
McFarlane et al. (2017)	Quant	Medicine, USA	*N* = 16 nurses (divided into four teams)	Use of smartwatches (with the HAIL-CAT program) to send messages to caregivers via smartwatch. This reduces the response time to clinically relevant alarms by a factor of three. No adverse effects on other tasks were reported. Trial users report positive assessments for usefulness, ease of use, requirements, and convenience.	O/EX
Connell et al. (2019)	Qual	Medicine	Interviews with *N* = 19 people from medical professions	Mobile result displays and automatic alarms show positive effects on patient care.	O/EX

Quant, quantitative study; Qual, qualitative study; review, review paper (systematic or unsystematic); Meta, meta-analysis; Concept, concept paper or opinion; Hand, handout, rule or information; EM, employees; L, leaders; T, teams; O, organization; EX, external support (e.g., IT staff or employees of a consultancy).

### 3.2. Inclusion and exclusion criteria

Due to the rapid development of ICT, the time frame of the search was limited to the period from 2000 to 2021. We included the following: (1) evaluation studies on concrete interventions; (2) scientific studies that allow conclusions to be drawn about design options for dealing with information overload; (3) studies on information quality; (4) studies on the design of work-relevant information to make it easier to process; (5) studies on the topic of knowledge management that might prevent information overload; (6) studies that give advice on the possible content of workshops at the individual level; and (7) studies that present examples of specific software for managing large amounts of information.

We excluded the following types of papers: (1) papers referring exclusively to multitasking and not simultaneously to information overload, (2) papers referring to the use of ICT outside of working hours or information overload when using social media, (3) technical papers on the possibilities of storing large amounts of data as well as purely technical-methodological presentations of ICT solutions; (4) papers on the area of consumer research and communication with customers, especially those on recommendation systems, and (5) papers giving design recommendations for specific occupational groups and application areas, such as librarians, molecular biologists, autonomous driving, studying and teaching, and medical knowledge from the perspective of patients (health literacy).

A total of 1,054 papers were extracted from the scientific databases, of which 72 were identified as suitable after screening. A total of 259 papers were extracted from the practice-oriented databases, of which 15 were identified as suitable and included in the review.

### 3.3. Intervention and design approaches

The causes of information overload can be found on the societal level as well as on the organizational and interpersonal levels. At the societal level, aspects such as the accelerated production of information and the rapid dissemination of information via the internet are causes of information overload. These societal aspects are difficult to change through occupational health management. At the organizational and interpersonal level, information overload can be caused by five different aspects (Eppler and Mengis, 2004): (1) information, (2) person, (3) tasks and processes, (4) organizational processes, and (5) ICT.

Information characteristics relevant to information overload include the quantity, frequency, intensity, and quality of the information. In addition, other general characteristics of the information may play a role (Eppler and Mengis, 2004). Indeed, Graf and Antoni (2020) report that the complexity of the information, its degree of ambiguity, the novelty of the information, and its structure are relevant to information overload.

At the person level, the person receiving, processing, or communicating the information is a relevant factor for information overload. Specifically, the person’s attitude, qualifications, or experience, including their competencies, skills, and motivation, can influence whether a given amount of information leads to information overload (Eppler and Mengis, 2004).

At the level of tasks and processes, information overload can arise from the work tasks themselves or from cooperation with other people. In particular, more routine procedures reduce the processing capacity required of the people involved, while more complex and new procedures are more likely to cause information overload (Eppler and Mengis, 2004).

Organizational processes refer to all formal and informal work structures at the organizational level (Eppler and Mengis, 2004). Changes and redesigns in organizational processes and team structures can increase the amount of information presented to the individuals, whereas standardized procedures or regulations can reduce the risk of information overload.

With regard to the level of information and communication technology, both *which* technologies are used and *how* they are used can be relevant to information overload (Eppler and Mengis, 2004). The emergence of new technologies and their use play an important role in causing information overload, as exemplified by widespread use of e-mail. It is important to take advantage of the opportunities offered by certain technologies, while at the same time minimizing the risks they pose.

These five levels of causes can serve as starting points for design options and interventions to address information overload. Accordingly, the results of this literature review are structured along these five levels. In this context, the design recommendations at the level of the person are classified as behavioral prevention measures, while the recommendations related to the other four levels can be regarded as structural prevention measures.

## 4. Results

### 4.1. Level of information

According to Graf and Antoni (2020), a distinction can be made between the quantity and the quality of information, and both aspects of information are related to information overload (Graf and Antoni, 2020). Quantity is mainly understood as the amount of information (objective). However, if the subjective perception of the amount of information is also taken into account, contradictory results can be obtained. For example, empirical studies suggest that there is a positive correlation between the quantity of information and information overload, but at the same time some studies have reported a negative correlation between these two variables (see Graf and Antoni, 2020). It can therefore be assumed that the subjective assessment of the quantity of information may be influenced by the available resources and the individual’s ability to manage the incoming information. The quality of information includes the various aspects that contribute to the fit of the information to the needs of the person receiving it. These aspects include, for example, the complexity or relevance of the information.

With regard to the quantity of information, it should be noted that a large number of papers have described the use of various information technologies to reduce the quantity of information. Therefore, papers that mainly describe and evaluate these technologies and thus address the problem of high information quantity are included in the section on information technology.

#### 4.1.1. Quality and presentation of information

Much of the literature on the presentation of information relates to the medical field. Indeed, the condition of critically ill patients can be affected if an important piece of information is overlooked, and time pressure is often high in medicine. Beasley et al. (2011) showed that problems of complex “information chaos” are particularly relevant in the medical field. Information overload is often cited as one aspect, along with information deficits, information conflicts, and scattered or incorrect information. The following papers each address one or more of these aspects.

Based on 17 primary studies, Khairat et al. (2018) reported in their review that visualization dashboards reduce the time spent collecting data, the difficulty of the data collection process, the cognitive load, the time to task completion, and the error rate. These visualization dashboards also improve situational awareness, adherence to evidence-based safety guidelines, ease of use, and navigation through the program. Therefore, the presentation of selected critical patient data in a clear manner can be recommended in clinical settings. Another review paper with the same target group was presented by Waller et al. (2019), and their results indicated that more than half of the included peer-reviewed primary studies (12 out of 22) on the implementation of dashboards showed positive effects on outcomes such as patient health, process outcomes, efficiency, and costs. However, the authors themselves reported that the empirical evidence on the effectiveness of dashboards implementation in clinical settings is limited by the low to moderate quality of the primary studies. It should also be noted that these studies rarely considered information overload as an outcome.

Three other original studies also address the clinical application context. Patapovas et al. (2013) tested a clinical decision support system, but since the study did not implement a pre-and post-survey and did not include a controlled-randomized design, the results only permit the conclusion that the test users used the electronic patient record regularly. Ahmed et al. (2011) compared a new format for representing information with an established format. With the new format of representation (reduction, filtering, and better categorization of patient data), the test subjects showed better performance, faster processing times, and most importantly also a lower task and information load. The study suggests that the reduction, filtering, and better categorization of patient data have a positive effect on reducing the effort required to process the information. Another dashboard for the clinical context was developed and tested by Tan et al. (2013) based on Toyota’s Andon Board. The dashboard extracts data from electronic patient records every minute and notifies clinicians of urgent orders (e.g., laboratory tests, medications, etc.), abnormal laboratory and radiology results, and infection alerts. Overall, the dashboard seems recommendable; in the study, it ensured efficient and safe work and was well accepted by the users. However, this empirical evidence is limited due to the lack of a control group.

In a different work context (an LED factory) but still with a similar approach, Wu et al. (2016) used eye tracking to investigate three different levels of complexity in the presentation of technical information at the human-machine interface. The results showed that the time it took the subjects to fixate on the target object differed significantly depending on the complexity of the presented information. Specifically, fixation was fastest in the low complexity condition. Another finding was that the search patterns of novices were significantly more complex than those of experts. Furthermore, experts reported lower cognitive load than novices. Overall, these findings suggest that user interfaces should be designed to be as simple and clear as possible while still providing the necessary level of functionality. Furthermore, the same amount of information may have different effects depending on the professional experience of the individual. Indeed, Ries and Deml (2019) reported a similar conclusion in relation to the work of control room staff; they also proposed specific recommendations on how to optimize the presentation of information in the form of video recordings.

Wnuk et al. (2016) proposed a way to visualize the content and progress of a project. This tool, called FSC+ (Feature Survival Chart), is specifically aimed at the fields of business management or project management. The tool makes it possible to visualize the scope of a project and to show the dwell time of individual project steps in the project, thus facilitating project decision making. A test of the tool with 20 sample users showed that the interviewees were positive about the tool. However, the learning curve for using the tool was longer than expected. The study showed that the tool is more useful for people who need to work with large amounts of data and information, while the tool is less useful for people who only need an overview of a limited number of project steps. The study provides weak evidence that the FSC+ tool can be used in project management and project leadership to reduce information overload. Roy et al. (2017) addressed the question of how to increase management’s knowledge of processes within the organization without leading to information overload. Based on an unsystematic review, the authors proposed the following solutions at the knowledge level: the use of summaries, comments by experts, indicators, and priority setting, the distribution of information over time, and the visualization of information. Solutions based on the use of digital media were as follows: the use of search engines, blogs, dashboards, news and updates, notes and virtual bookmarks, and alerts. In addition, with the aim of providing relevant information to decision makers in companies, Dzokoto et al. (2013) proposed a “SMART Push System” based on a literature review. This system actively suggests the relevant documents or content to decision-makers, taking into account all the available information and contextual knowledge, without them having to actively request the information. However, this article was only a conceptual work.

Riener and Ferscha (2008) took a different approach, initially independent of the work context. They proposed the use of tactile stimuli to relieve the visual and auditory sensory channels. Their study focused exclusively on spatial orientation and tested the use of a vibrotactile belt to inform subjects about the spatial distance to a target object. Based on the results of the study, we cautiously conclude that it may be useful to use tactile stimuli to convey information. However, the participants only showed a learning effect when they were informed about the accuracy of their distance estimations, whereas their estimation performance deteriorated when no feedback was provided.

#### 4.1.2. Quantity of information

Shah et al. (2019) examined the quantity of information sent to primary care providers through the Department of Veterans Affairs in the US. They also looked at new regulations to filter the information sent, as well as staff training on how to individually enable and disable certain types of messages. The study showed that the number of daily messages could be reduced with these policies, but no concrete impact or evaluation of the staff training was reported. It should also be noted that there was no control group and that the implementation of the intervention varied between regions.

Another study in the clinical work context by Pickering et al. (2013) examined the information used by intensive care unit (ICU) staff when a new patient is admitted. The aim of the study was to identify the type of information that needs to be prioritized in the ICU. To answer this question, observations and interviews were conducted over 1.5 years in three different ICUs. Out of a total of 51 different clinical information concepts, an average of 11 concepts were used when a single patient was admitted. The four most commonly used concepts were heart rate, oxygen saturation, respiration, and blood pressure, and each was used in more than half of the admissions. The study suggests that when a patient is transferred to the ICU, certain information should be presented in a prioritized manner. Access to other information should not be prevented, but the most commonly used categories of information should be presented in a clear and concise manner.

Another approach to reducing the amount of information or helping individuals remember the most important information was examined in a Cochrane systematic review by Arditi et al. (2017). They focused on computer-generated reminders provided in paper to healthcare professionals. The data from 34 individual studies showed that this form of intervention significantly improved the quality of care. There was also weak evidence that patient outcomes could be positively influenced by these reminders. However, the extent to which this intervention could reduce information overload for health professionals was not investigated.

#### 4.1.3. Summary: level of information

Empirical evidence on the quantity and quality of information to reduce information overload focuses mainly on the digital representation of information. When designing software, it is important to ensure that the amount of information presented is manageable and customizable. At the same time, in the software, it should be possible to intuitively access background information that is not visible at first glance. Design principles and design laws should also be considered. Although the studies to date are of relatively moderate quality (e.g., few have used a randomized control group design) and mainly relate to the medical field, the findings are also applicable to other professional groups for whom it is essential to have important information presented as clearly as possible (e.g., employees in control rooms or managers). Based on the literature presented here, the use of dashboards appears to be recommended.

### 4.2. Person level

At the individual level, the literature included in this review offers some advice on how to manage information and information overload in general. Specifically, information overload can be addressed at the personal level through education. Furthermore, we present recommendations on how to deal with e-mail and the Internet in general. The following section also includes suggestions on how to actively manage the boundaries between work and leisure time and the workplace itself.

#### 4.2.1. Dealing with information overload

Information overload should be recognized as early as possible (Mustapar et al., 2016) and proactively addressed at the individual level (Landale, 2007). Landale (2007) presented a three-step approach that can be applied by individuals in combination with memory training: (1) receive and evaluate the document and decide whether to read it, delegate it, or ignore it, (2) gain an initial understanding of the document and decide whether to read it in detail and, if so, how quickly and how deeply, (3) adapt the new knowledge.

Similarly, Warbington (2000) recommended that when receiving information, one should first clarify the goal one is trying to achieve, from which the importance of different pieces of information can be assessed. Accordingly, a survey of 124 managers by Farhoomand and Drury (2002) and an editorial by Kluge et al. (2020) suggest that filtering information is seen as a helpful measure against information overload. This filtering can be achieved, for example, by suppressing, deleting, or selecting irrelevant information (Kluge et al., 2020). In the process of filtering or screening, according to Farhoomand and Drury (2002), delegating work is also an option, and this delegation should be directed not only to colleagues but also, if possible, to digital agents (Kluge et al., 2020). Finally, prioritizing information can also be a helpful action to manage information overload (Farhoomand and Drury, 2002).

Mindfulness also plays a role in managing information overload, and according to an unsystematic review by Stich et al. (2018), mindfulness skills should be developed in order to manage e-mail-related information overload. Mindfulness can be assessed and improved through questionnaires that measure and provide feedback on the level of strain experienced by information overload or attitudes toward ICT.

#### 4.2.2. Training

At the individual level, previous studies have recommended attending training to reduce and prevent information overload (e.g., Drössler et al., 2018; Stadin et al., 2020), with companies in particular being encouraged to invest in such training for their employees. However, Pfaffinger et al. (2020) also suggest that individuals should proactively demand further training opportunities.

A review by Antoni and Ellwart (2017) made it clear that individuals are able to process a large amount of complex information if they have the competencies to use modern ICT. These skills should be trained through further education, for example, on the functionalities of these technologies. Furthermore, according to Nagarajah (2016), the introduction of new technologies should be accompanied by adequate training in order to prevent technostress.

Specifically, in addition to the training in digital skills (Drössler et al., 2018; Lehman and Miller, 2020), the literature recommends improving media and information literacy (Bundesanstalt für Arbeitsschutz und Arbeitsmedizin (BAuA), 2006; Benselin and Ragsdell, 2016; Drössler et al., 2018). With regard to the use of software, this literacy includes, for example, learning helpful but often unknown functions of the software (Bundesanstalt für Arbeitsschutz und Arbeitsmedizin (BAuA), 2006).

Based on a case study, Cheuk (2008) also suggested that information literacy is a human resource development issue in four dimensions: first, the skillful use of information at a strategic level; second, the organization and control of information; third, the knowledge of access and tools; and fourth, the linking of finding and using information.

In addition, individuals should be taught appropriate work strategies to deal with information overload, such as how to manage large amounts of information (Bundesanstalt für Arbeitsschutz und Arbeitsmedizin (BAuA), 2006). These strategies could include improving self-and time management, as these are effective measures against information overload (Bundesanstalt für Arbeitsschutz und Arbeitsmedizin (BAuA), 2006; Drössler et al., 2018).

According to a quantitative study by Yener et al. (2020), in addition to training in time management, technological self-efficacy training could also counteract the negative effects of technostress on burnout and performance. Mahapatra and Pati (2018) highlighted that techno-invasion and techno-insecurity are also positively related to burnout, while the latter mediates the relationship between techno-complexity and burnout. Therefore, techno-insecurity and perceived techno-complexity should also be addressed in training measures. The study by Le Roux and Botha (2021) provides a complementary reference to these findings, as their work showed that perceived techno-complexity and techno-uncertainty increase with age. Consequently, training in this area should be provided to older employees in particular.

Zhao et al. (2020) suggested further avenues for interventions to address information overload. First, problem-focused coping (e.g., performing a cognitive assessment of technostress and seeking instrumental support) is helpful in alleviating information overload. Second, training in emotion-focused coping or venting is also recommended. Based on this, Becker et al. (2021) found in a quantitative study with 3,363 knowledge workers from Germany that coping strategies reduced the negative indirect relationship between technostress and performance mediated by exhaustion. Therefore, the authors recommend that different coping strategies should be trained. Although both dysfunctional (avoidance of the problem) and active-functional (including problem-focused and emotion-focused coping) coping strategies reduce the strain caused by techno-stressors, the negative long-term consequences of dysfunctional coping should be explained to individuals, and in particular, active-functional strategies should be part of further training measures.

A cross-sectional study by Yin et al. (2018) focused on the timeliness of information processing, which refers to the perceived support for timely information provided by mobile ICT. Their study found that the timeliness of information processing reinforces the negative relationship between information overload and job satisfaction and is therefore not recommended as a coping mechanism.

Based on their qualitative and quantitative study results, Moser et al. (2002) developed an exemplary training concept to be applied at the personal level, including the following aspects: know-how, efficient handling of information overload, time management methods, and training on e-mail program. The training was highly accepted by the participants, led to an increase in knowledge, and reduced the participants’ feeling of information overload.

In a quantitative intervention study, Soucek and Moser (2010) examined a training intervention to explain the use of modern ICT, and their results showed that e-mail communication could be improved by training in media literacy, individual work processes, and knowledge of the principles of e-mail communication. According to Bundesanstalt für Arbeitsschutz und Arbeitsmedizin (BAuA) (2006) and Stich et al. (2018), training-based interventions in relation to e-mail use should also include “netiquette,” (i.e., topics such as virtual politeness, respect, and commitment). With regard to training on e-mail use, Antoni and Ellwart (2017) further noted that individuals should be made aware of the negative effects of e-mail interruptions and should be presented with concrete techniques for managing e-mails efficiently. For example, setting fixed times for processing e-mails can be helpful.

A concrete example of an intervention that has already been developed and evaluated is the training program of the initiative “New Quality of Work,” which includes modules on the organizing of incoming mail, processing and managing e-mails, and designing correspondence (Bundesanstalt für Arbeitsschutz und Arbeitsmedizin (BAuA), 2006). According to Moser and Soucek (2005), a needs analysis is required before a training intervention should be offered in a company. Ultimately, a shared e-mail culture can be developed through joint participation in the program.

De Bruin et al. (2020) showed a positive effect of mindfulness training with regard to stress (measured by the Perceived Stress Scale). However, a positive effect of mindfulness training specifically on information overload cannot be inferred from these findings. According to an interview study by Camargo (2008), other creative solutions, such as learning videos or presentations, can also contribute to the further training of employees in relation to managing information overload.

#### 4.2.3. Dealing with E-mails

According to Drössler et al. (2018), managing e-mails poses a challenge for employees in terms of information overload. Indeed, both individuals and organizations should take responsibility for implementing measures to address this issue, which can be done by creating a detailed information culture in the company and clear internal company regulations, such as communication and e-mail guidelines (Bundesanstalt für Arbeitsschutz und Arbeitsmedizin (BAuA), 2006; Ramsay and Renaud, 2012; Drössler et al., 2018; Stich et al., 2018; Stadin et al., 2020). Soucek (2009) also recommended structuring and managing the inbox, scheduling e-mail communication, and using e-mail correspondence in a manner appropriate to the medium.

Clear articulation of concerns related to task objectives is considered important in e-mail communication. Furthermore, in order to avoid unnecessary communication, it should be made clear whether further action is required as a result of the e-mail sent and, if so, what form this action is expected to take (e.g., how quickly a response is expected; Bundesanstalt für Arbeitsschutz und Arbeitsmedizin (BAuA), 2011; Ramsay and Renaud, 2012). In addition, the necessity of the information should be questioned before sending an e-mail communication, and e-mails should only be sent to relevant recipients (Rack et al., 2011). Standard templates can be used to reduce the effort required to formulate e-mails (Bundesanstalt für Arbeitsschutz und Arbeitsmedizin (BAuA), 2011). Furthermore, the group of people to whom the information is disseminated to should be limited by avoiding the use of carbon copies (CC) and forwarding e-mails (Bundesanstalt für Arbeitsschutz und Arbeitsmedizin (BAuA), 2011; Ramsay and Renaud, 2012; Drössler et al., 2018), the amount of information disseminated by the e-mail should be kept as small as possible, and large attachments should be avoided (Bundesanstalt für Arbeitsschutz und Arbeitsmedizin (BAuA), 2011). So-called “buck-passing,” (i.e., passing on a task to another person by forwarding an e-mail, should be avoided (Ramsay and Renaud, 2012), but blind copying (BCC) should be retained if deemed appropriate, as there are usually good reasons for using this functionality. However, duplicate communication through multiple channels should be avoided according to Drössler et al. (2018). Individuals should refrain from regular follow-up e-mails (Ramsay and Renaud, 2012) and instead consider other methods of communication, such as face-to-face contact instead of e-mail (Camargo, 2008; Bundesanstalt für Arbeitsschutz und Arbeitsmedizin (BAuA), 2011; Drössler et al., 2018). In addition to individual strategies, such as skimming (reading quickly to get a general overview), automated filters in the inbox can also be helpful for recipients of large amounts of e-mail information (Rack et al., 2011). Indeed, developing rules and filters and integrating them into one’s e-mail use can help separate important e-mails from irrelevant information, such as spam (Camargo, 2008). Ramsay and Renaud (2012) recommend ignoring or deleting potentially risky e-mails as a measure against information overload. This approach is particularly useful when virus-laden content is suspected.

To counteract the accumulation of information, subscriptions to automated services such as newsletters or newsfeeds should be made selectively (Drössler et al., 2018). Similarly, individuals should also inform their colleagues directly if they do not need information sent via their e-mail distribution lists (Camargo, 2008).

To protect individuals from compulsive e-mail checking, Ramsay and Renaud (2012) recommended two strategies. First, colleagues should accept that individuals have different ways of managing e-mails in terms of how often they read them. Second, individuals should free themselves from the expectation that e-mails should be answered outside of work hours, thus allowing them to make a clear distinction between work and private life (*cf.* Camargo, 2008).

#### 4.2.4. Dealing with the internet

A report by the Bundesanstalt für Arbeitsschutz und Arbeitsmedizin (BAuA) (2011) provides concrete recommendations on how to manage the Internet in general. In particular, the use of meaningful and concise search terms is recommended to significantly reduce the number of search results. In addition, the topics of documents and materials from the internet should be checked immediately to eliminate information that is not relevant. The basic principle of these recommendations is to identify only as many documents on a topic as can be processed in a reasonable amount of time. According to the authors, a clear filing system can help navigate through the information. Furthermore, when working with the Internet, direct communication with colleagues about the perceived importance and relevance of various documents can also reduce one’s own information overload (Bundesanstalt für Arbeitsschutz und Arbeitsmedizin (BAuA), 2011). Finally, a systematic review by Drössler et al. (2018) suggested that active information searches should be used instead of push notifications.

#### 4.2.5. Active design of the workplace

Pfaffinger et al. (2020) report that active workplace design and boundary management can be helpful against information overload. In fact, an interview study by Camargo (2008) with 17 employees in the “high technology” sector also found that separating work and private life can reduce the stress caused by e-mails.

Soucek and Moser (2010) recommended self-management techniques, such as prioritizing and sequencing tasks according to one’s needs, to improve the individual workflows. For example, managing e-mails should be integrated into an individual’s daily schedule at a time that makes sense for them.

Bundesanstalt für Arbeitsschutz und Arbeitsmedizin (BAuA), (2011) recommends actively creating time for breaks and reducing interruptions during these times, which can be achieved, for example, by switching off the incoming notifications for e-mails (*cf.* Drössler et al., 2018). Finally, to reduce technostress, no e-mails should be processed or sent outside of working hours (Gaudioso et al., 2017).

#### 4.2.6. Summary: person level

**Figure 2:**
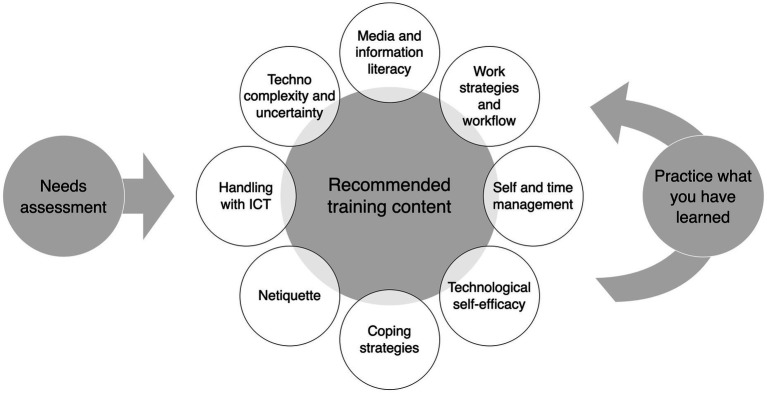
Results of the systematic review on content of training.

At the individual level, several studies have recommended that individuals participate in training interventions that, in addition to supporting the development of general competencies such as self and time management, should improve their competencies in managing ICT and the software used in the company. However, there is a lack of methodologically sound empirical studies that test the effectiveness of the proposed intervention approaches. In fact, we identified only two evaluated interventions that reduced subjective information overload among participants, but these studies did not include any objective measures. In terms of avoiding information overload, e-mail management seems to be particularly challenging. In addition to participating in training, studies have suggested concrete actions that individuals can take to counteract information overload. An example is to organize one’s workplace in a way that supports the setting and maintaining of boundaries between work and private life and creates free spaces and breaks. It should be noted that while the advice for individuals is presented at the individual level, the support of the company in the implementation of these strategies is elementary. For example, the company’s regulations, the corporate culture, or human resource development policies may be important in helping employees manage information overload. Figure 2 provides an overview of the suggested content for training.

### 4.3. Level of tasks and processes

At the task and process level, it may be useful to standardize certain procedures to address information overload (Eppler and Mengis, 2004). Piecha and Hacker (2020) and Piecha (2021) presented a detailed report on a multi-year multi-method research project on information overload. The aim of the report was to identify approaches to managing a large amount of information transmitted through ICT at work in a non-overwhelming way. From their systematic field studies, the authors derived several recommendations. These include the needs-based design of the media landscape, the regulation of organizational information flows, the adequate measurement of time when working with digital media, the reduction of parallelism and diversity of tasks, the development of employees and managers, and the individual planning of work. In addition, Stadin et al. (2020) interviewed 20 health care managers in Sweden using the critical incident method. The negative aspects of digital communication mentioned were high workload, the invasion of privacy, and negative feelings (e.g., fear of missing something in the e-mail inbox). Promoting digital literacy, designing ICT according to needs, and redistributing work and ICT systems were mentioned as possible solutions to the negative aspects of digital communication. Furthermore, e-mail culture, support from colleagues, individual resources (individual strategies, such as routines and structures), individual competencies (e.g., learning by doing or preparation), and organizational resources (e.g., IT support) were mentioned as important aspects in dealing with information overload.

Stich et al. (2018) highlighted the negative consequences of excessive use of digital information technology and ICT: technology overload, interruptions, multitasking, work-home interference, and cyber deviance. The following process recommendations were made to address these issues: (1) establish clear policies on ICT use (e.g., blocking e-mail traffic outside working hours, switching from e-mail to an internal social network, establishing policies against cyberbullying or regarding ICT use in general); (2) implement individual interventions; (3) create individual mindfulness (e.g., measure and provide feedback on the level of technostress or attitude toward ICT through surveys); (4) create mindfulness toward colleagues (particular responsibility of managers, but colleagues should also be asked which communication channels they prefer).

Bordi et al. (2017) introduced the term information ergonomics, focusing on the workload induced by information-intensive tasks. In their study, discussions in several focus groups with a total of 36 employees (including insurance and financial service providers) were qualitatively evaluated. The following aspects were identified in the discussions: the establishment of common rules (etiquette), quiet times, training in the use of tools, and prioritization.

In their editorial, Kluge et al. (2020) discussed the filtering of information as a measure to address information overload. They also emphasized that tasks can be delegated from humans to digital agents and that management information systems and interactive assistance systems can be used to reduce information overload. Effective measures also include “organizational unlearning,” which refers to forgetting information that is no longer relevant.

Okolo et al. (2018) conducted a cross-sectional study with 319 bank employees in Nigeria to examine the indirect relationship between job-related resources according to the Job Characteristics Model (i.e., task feedback, task autonomy, task significance, task identity, and skill variety) and work engagement mediated by the experience of technostress. Technostressors (overload, complexity, and invasion) could be categorized as challenging demands, defined as a demand that shows motivational effects in addition to being straining. Activity resources showed a positive indirect relationship with work engagement mediated by technostress. Concrete recommendations for action cannot be derived from this study, but the indication that experienced technostress may have the potential to act as a challenge suggests that motivational gains may be derived from technostress under certain conditions.

Oldroyd and Morris (2012) focused on a specific group of employees, the so-called high performers or stars, whose productivity can be up to 10 times higher than the average in an occupational field. Such stars tend to be very well connected socially, which means that the amount of communication and, thus, the information load they experience is very high. To maintain the productivity of such stars, the authors suggest, among other things, (1) training in the ability to distinguish between useful and superfluous information, (2) the formation of schemas to support long-term memory, (3) decision latitude, (4) supportive colleagues, (5) reducing the breadth of the social network while increasing the density, and (6) distributing standardized information to the entire network instead of individualized information. The authors also made other recommendations at the organizational level: (1) clarify where information can be found, (2) increase the cost of sharing information, (3) use information systems to contextualize information and make it more useful to employees; (4) create special knowledge-sharing positions for star employees, and (5) encourage relationships with other star employees. Some of these recommendations do not appear to be limited to star employees.

#### 4.3.1. Cooperation in teams

Ellwart et al., 2015 evaluated an online intervention for structuring teamwork (STROTA), which was shown to reduce information overload. In the STROTA intervention, team members are first encouraged to analyze the current situation with regard to the triggers and conditions of experienced information overload in a structured way. Then, a facilitated team discussion creates a shared mental model and situational awareness (team situation awareness), and the team develops concrete goals and plans (team adaptation).

Based on interviews with 10 emergency responders with leadership roles, Norri-Sederholm et al. (2015) also emphasized the importance of situational awareness, which depends on having relevant information available. Paul and Nazareth (2010) used a group support system to regulate the flow of information within a team, helping them to process information effectively despite high complexity. As an intervention, the groups were given access to aggregated information from the work of previous groups engaged in a similar decision situation. In terms of the results, the provision of a decision scheme cannot be unconditionally recommended, as the expected effect (a higher threshold for the occurrence of information overload) did not occur. Instead, the relationship between information complexity, time pressure, and time to decision changed fundamentally to a U-shaped relationship in the treatment group. Specifically, at high levels of information complexity, the decision time was longer when the decision scheme was available to the group. Therefore, the advantages of having a schematic summary of information are only relevant for information of medium complexity.

Wang et al. (2007) investigated how an adaptive awareness system for shared virtual workspaces can be technically implemented and how its comprehensibility and usefulness can be evaluated by testers. The goal of this study was to present a system to reduce the information load of digital collaboration. However, this study provides only weak evidence that the automatic adjustment of notification rules can be useful in virtual collaboration, because information load and information overload were not considered as outcomes.

The results of a study by Bergström et al. (2010) suggest that applying the theoretical basis of the coordination of joint activities is a promising way to develop a contrasting perspective of the factors that allow teams to maintain control in escalating situations. In a simulation in this study, both lay and expert teams were confronted with an escalating emergency situation on a ship. A key finding of the work was that in such highly complex situations information must be prioritized and filtered to avoid information overload.

Ferreira et al. (2011) also aimed to ‘address the problem of information overload in synchronous group work’ (p. 643). The authors experimentally examined team performance in a brainstorming task under the conditions “without attention management” (one team member’s ideas were sent directly to all the group members) and “with attention management” (team information was sent only when the team member was not currently working individually). The use of attention management resulted in better team performance in terms of the number of ideas generated, although the generalizability to other types of team tasks is unclear.

Stich et al. (2018) found that creating mindfulness toward colleagues can be helpful in reducing information overload, for example, inquiring about which communication channels colleagues prefer. The authors suggested that leaders should take responsibility for this collegial mindfulness. In meetings, it can be helpful to establish rules for the use of digital media, such as turning off notification signals during meetings (Drössler et al., 2018).

#### 4.3.2. Leadership

Only a few papers have reported suggestions for leaders to reduce information overload. For example, Becker (2009) conducted interviews with 12 managers in the health care sector, and based on the results, the author suggested limiting organizational priorities, creating clarity about project responsibilities and decision-making, organizing fewer and better-structured meetings, and establishing guidelines for the use of technology.

Spagnoli et al. (2020) reported that workaholism and authoritarian leadership were positively correlated with experienced technostress. Specifically, for employees who worked exclusively from home, authoritarian leadership strengthened the relationship between workaholism and technostress.

Based on her own experience as a trainer and coach, Mill (2010) presented ideas and strategies for managers to introduce training and other measures to reduce work stressors related to information overload and ICT use and to promote a positive work culture. In her view, these strategies should include effective communication, training for employee development, coaching, mentoring, and good leadership and inspiration, which can all positively influence the work culture and, thus, lead to improved health, and well-being.

#### 4.3.3. Summary: level of tasks and processes

In general, measures for stress-optimized work design have been proposed for the area of tasks and processes. Information overload is often associated with other quantitative and qualitative stressors, such as time pressure, high workload, interruptions, or role ambiguity. Although the causal direction of these relationships is not clear, a reduction in the accompanying stressors seems to contribute to a reduction in perceived information overload or technostress. Similar to the personal level, many proposals at the task and process level include approaches to increase the competence of individuals in dealing with digital information technologies. Additional support services (IT support, supervision) are also seen as helpful. The establishment of common rules (etiquette in dealing with ICT) has been mentioned several times in the literature as a worthwhile approach to reduce information overload. At the team level, the establishment of situational awareness (attentiveness to the situation) and shared mental models have been suggested. In addition, assistance systems for filtering and prioritizing information have been mentioned as starting points for dealing with information overload. Recommendations regarding leadership remain relatively superficial; a supportive and constructive leadership style is considered favorable, whereas an authoritarian or destructive leadership style tends to be unfavorable with respect to technostress and information overload.

### 4.4. Level of the design of organizational processes

At the level of organizational process design, behavioral and action guidelines can be introduced to address information overload (Manwani et al., 2001). The strategic use of internal communication can reduce the risk of information overload (Remund and Aikat, 2012). However, corporate culture is also a relevant factor (Sumecki et al., 2011). Kluge and Gronau (2018) investigated the process of intentional forgetting to reduce information overload in change processes. Furthermore, Day et al. (2012) showed that the provision of up-to-date software and personal support from IT staff can mitigate the negative effects of information overload.

Farhoomand and Drury (2002) interviewed 124 managers in different countries about their experiences and approaches to information overload. Among other things, the managers mentioned the following: 1) identifying the dimensions of information overload (extent, irrelevant information, time constraints, variety of information channels) to provide targeted support to affected employees; 2) in order to provide better tools and techniques to better process information from internal and external sources; and 3) ensuring flatter hierarchies in combination with intelligent IT within the organizational structure.

In a cross-sectional questionnaire study, Florkowski (2019) examined the associations between organizational policies and technology-related stress experiences, uncertainty, and job satisfaction in a sample of managers from 169 human resources (HR) departments in the US. The managers highlighted the importance of expectation management in the use of technology in human resources (HR) and recommended the continuous adaptation of the organizational climate in terms of flexibility, risk-taking, evidence-based experimentation, and support for innovation.

Gaudioso et al. (2017) tested a serial mediation model and showed the indirect effects of techno-invasion via work–family conflict and of techno-overload via distress and coping (adaptive vs. maladaptive) on exhaustion. However, the cross-sectional design is a major limitation of this study. From the reported findings, the authors derived their recommendations to eliminate maladaptive coping strategies and subsequently develop adaptive coping strategies. In addition, they reported that awareness of technology use should be increased and barriers to technology use should be removed (e.g., through training, work design, reward systems, peer pressure, or technical support). To reduce technostress, the authors recommended, among other things, not sending and processing e-mails outside working hours and improving the organizational culture.

Based on interviews with 26 employees from different countries, Pfaffinger et al. (2020) suggested the following to reduce information overload: invest in IT security, communicate, structure, and accompany the introduction of new technologies effectively, develop new work concepts, clarify expectations regarding flexibility of time and place, ensure compliance with work regulations, implement ergonomics in the home office, provide the latest technologies and corresponding technical support, provide a helpdesk, and training for managers, and provide training for individual employees. In addition, the study also addressed the societal level with the following recommendations: ensure participation, public training, or help desks, ensure high speed Internet for mobile phones, prohibit or regulate surveillance, protect data rights, enact penalties for violations, offer social services and security in case of job loss, ensure the humanity of new forms of work, create a legal framework for the separation of work and private life, and provide a functioning infrastructure for companies.

Soucek (2017) examined the extent to which e-mail communication agreements are perceived as useful in organizations. As a conclusion, Soucek (2017) explained that “such agreements on e-mail communication are mainly adopted company-wide and outweigh the number of intra-departmental agreements, with the intra-departmental agreements proving to be particularly helpful” (Soucek, 2017, p. 20). Stich et al. (2018) also recommended setting clear guidelines for the use of ICT. For example, they suggested that e-mail traffic should be blocked outside working hours, that switching from e-mail programs to an internal company social network may be helpful, and that policies against cyberbullying or more generally about the use of ICT should be established.

#### 4.4.1. Summary: level of design of organizational processes

Recommendations at the level of organizational processes cannot always be clearly distinguished from those at the level of tasks and processes. Indeed, the (participatory) development of policies and the establishment of a corporate culture regarding the use of ICT are also addressed in the studies at this level. In addition, there are references at this level to technical assistance systems and to the promotion of competence in the use of digital technologies in the context of human resources development measures.

### 4.5. Information technology level

Media richness theory suggests that the choice of an appropriate technology is relevant for the transmission of information (Graf and Antoni, 2020). At the level of information technology, the implementation of filters appears in several papers. Other technology-related approaches can be grouped under the categories of algorithms that summarize or extract information and the automation of monitoring tasks. Monitoring tasks can be considered as a specific form of work task, which is usually characterized by a large amount of information. However, this section first presents some of the results that relate more generally to the use of ICT.

#### 4.5.1. Use of information and communication technology

Based on a cross-sectional study by Ayyagari et al. (2011), it can be concluded that caution is required when introducing new ICT or changing existing ICT. Dynamic changes in ICT should be considered a source of stress. Drössler et al. (2018) warned that the introduction of a new technology should be thoroughly considered and, based on this, special care should be taken when choosing a form of ICT for the corresponding information transfer. Similarly, in line with media richness theory, Kauffeld et al. (2016) argued that the richness of the chosen medium should match the type of information to be transferred. For example, when establishing a relationship with a new colleague, face-to-face communication would be preferred, because it is the richest form of communication, whereas the coordination of an appointment could be clarified with the least rich form of information—an e-mail. Regarding the appropriate choice of ICT, Oehme et al. (2019) showed that the use of ICT in control rooms and in the work of field staff has a high potential to facilitate team processes and reduce errors. In addition, previous literature has recommended the use of filtering capabilities. Warren (2014) made other recommendations, such as the use of unified file systems, tagging, context management, and the use of semantic technology to combat information overload, facilitate context switching, and help employees integrate information.

Tagging (tags are used to add information to content; tagging is primarily used to make information easier to find or link) was recommended by Jackson and Smith (2012). In their qualitative case study, the authors showed that traditional hierarchical file systems can lead to the retrieval of irrelevant or no information, even though the relevant information is present. The study provides an initial indication that tagging could provide an effective solution to this problem and reduce the retrieval of duplicate or irrelevant information.

Demirsoy and Petersen (2018) proposed an example of semantic technology and evaluated this using an interview study. The proposed semantic knowledge management system enables automated text processing. Specifically, a basic ontology has to be created, which is then filled by automated semantic annotation in the next step. In a final step, the users are provided with a convenient and efficient keyword search (Demirsoy and Petersen, 2018) to prevent information overload caused by a large amount of information. Although the system was positively evaluated by the users in this study, information overload was not considered as an outcome.

A nonsystematic review by Celi et al. (2014) provides a variety of recommendations for the design of data systems in medical practice to prevent information overload. The authors provided ten suggestions for the design and implementation of a medical data system: 1) automatic integration of the data, 2) collection and integration of newly available and historical data, 3) automatic suggestion of diagnostic, therapeutic, prognostic, and other documentation suggestions, 4) the use of machine learning, 5) offering individual customization options, 6) ensuring aggregation of the data for research, 7) considering reports with “best practice” recommendations, 8) ensuring changeability of the system, 9) changeability also regarding to the integration of new data, and 10) prototype testing.

Martignene et al. (2020) proposed an R-based tool that extracts the relevant information from patient data and presents it visually. The program is called Heimdall and was initially evaluated only in terms of its functionality. The field application of this tool is still pending.

According to Fellmann et al. (2019), the implementation of stress-sensitive IT systems would be useful in principle, but urgent attention must be paid to the needs of the users. For example, in their study, the users clearly preferred a two-step process, in which, initially, they were first informed about their workload and stress, while recommendations for countermeasures were presented only upon request.

#### 4.5.2. Filter and decision support systems

In a vignette study, Ulfert et al. (2022) examined decision support systems with varying degrees of autonomy. The effects of the experimental conditions on participants’ information overload and their intention to use the system were considered as outcomes. The study found that increasing the degree of autonomy of the decision support system (under certain conditions) led to a reduction in information load. However, respondents still reported high levels of technostress, and increasing automation had a negative effect on their intention to use the system.

A similar decision support system was proposed by Garnsworthy et al. (2004) as a prototype for the work of metro surveillance staff. In addition, according to Mack et al. (2009), decision support systems can improve clinical work in pediatric ICUs. However, the choice of system should be made with careful consideration of the potential negative effects of introducing a new technological system.

#### 4.5.3. Algorithms for summarizing or extracting information

Sappelli et al. (2016) proposed an algorithm that goes beyond the filtering and categorization of traditional e-mail program systems. In fact, this algorithm can extract the action performed by the sender (e.g., reply, deliver, greet, etc.), the required response (e.g., no reply required, immediate reply, etc.), the sender’s implicit reason for the e-mail (e.g., internal collaboration, travel planning, etc.), and the number of tasks contained in the e-mail. In addition, Zhang and Xiao (2018) proposed another technology developed to increase the speed of text comprehension.

#### 4.5.4. Automating monitoring tasks

One area where employees are exposed to too much information at once is camera surveillance. Therefore, Tung et al. (2011) developed an algorithm that detects unusual movement patterns from surveillance camera images and alerts employees of conspicuous or suspicious cases. In their study the new algorithm was tested only on sample data, without measuring any attitudinal or behavioral data. The NoDoze program (Hassan et al., 2019) can be applied to IT security. The algorithm ranks incoming security alerts according to their urgency, with potential false alerts being ranked very low. This algorithm is intended to counteract “threat alert fatigue” among IT staff. Another algorithm based on traffic monitoring has been proposed with BeAware (Baumgartner et al., 2014). As these three studies only focused on the functionality and accuracy of the algorithms, no conclusion can be drawn as to whether these algorithms reduce the information overload of (IT) security staff.

McFarlane et al. (2017) tested the transfer of a military surveillance system (HAIL) to a clinical setting. Using the system, nurses responded three times faster to clinically relevant alarms, with no negative impact on their other tasks. On a subjective level, the test users indicated that they found the system useful and comfortable. Therefore, the study provides initial evidence that the use of the HAIL system in a clinical context could be a starting point for reducing the amount of information presented to clinical staff. With a similar aim, Connell et al. (2019) also tested the use of automated alarms in medical care and showed that patient care improved due to the alarms. However, effects on information overload were not reported. For the implementation of automated alarms, the authors recommended that users should be trained to ensure that the workload and the handling of the information received is well organized. Furthermore, such systems should be continuously optimized to reduce false alarms (Connell et al., 2019).

#### 4.5.5. Summary: use of information and communication technology

A wide variety of technological options have been proposed in the literature to reduce the risk of information overload. Overall, user needs must be considered when introducing new technologies to minimize the likelihood of increasing technostress. Specific suggestions for reducing information overload when using ICT include the use of tagging to complement traditional folder structures, filters and decision support systems, and algorithms for automatically summarizing text to reduce the amount of information presented. In evaluating these recommendations, it is important to keep in mind that many of the technological options have not been systematically evaluated in human use. However, studies that have evaluated these interventions suggest that care must be taken to ensure that the technology does not unduly restrict the user’s autonomy.

## 5. Discussion

**Figure 3:**
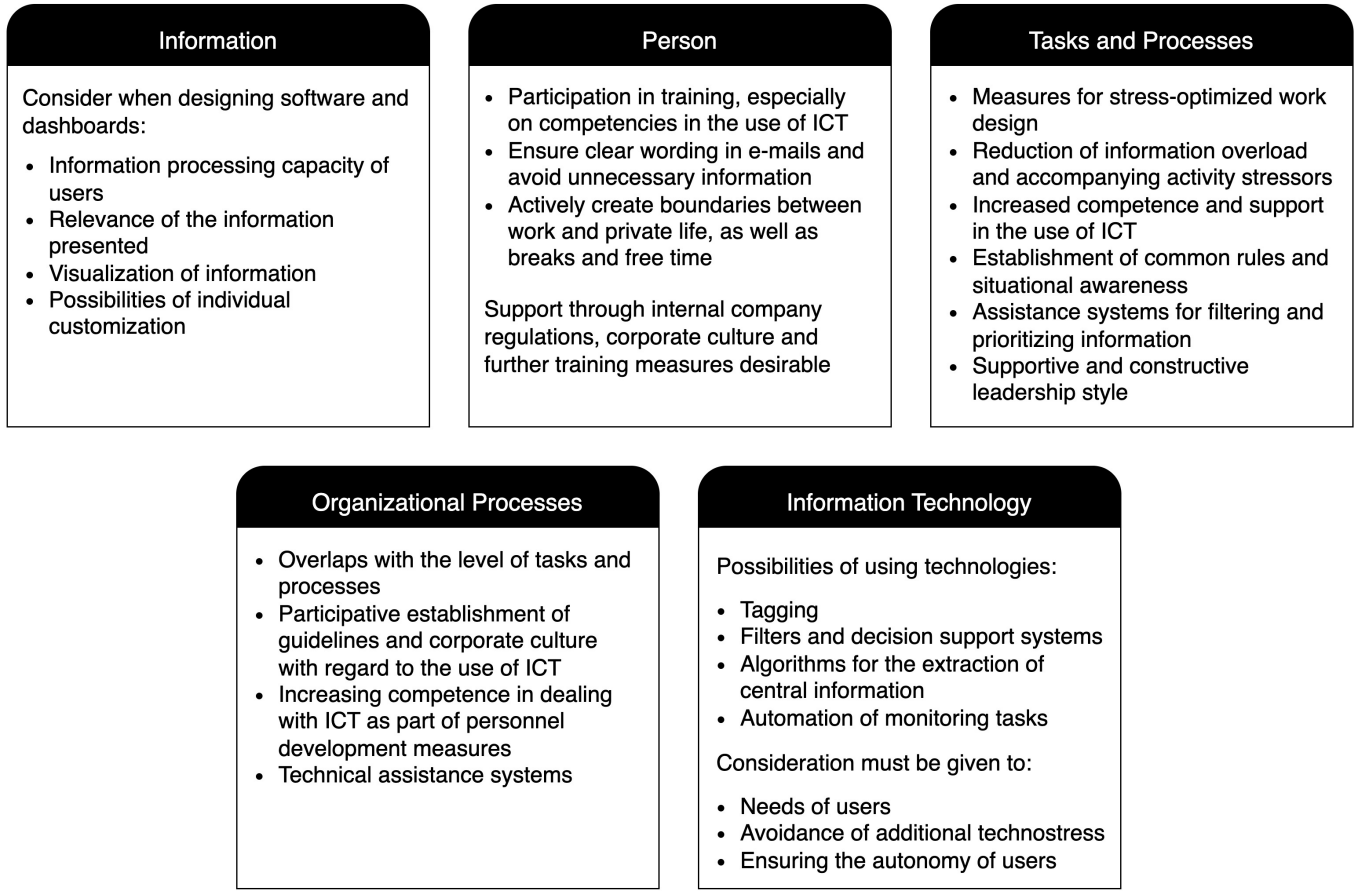
Summary of the key results of the systematic review.

Most empirical research in the area of information overload has focused on examining predictors and consequences or providing descriptive data on the prevalence of information overload. Some of this research can be used indirectly to derive recommendations for workplace interventions. However, the focus of this report is on concrete measures for managing large amounts of information in the work context. The following sections summarize the collected recommendations, and Figure 3 provides an overview of the key findings.

### 5.1. Summary of the results across the five levels

At the level of information design, several studies were found, which can be roughly divided into those focusing on the quality of information and those focusing on the quantity of information. Regarding the quality of information presentation, most studies have focused on the medical field. Specifically, visualization dashboards have been recommended to reduce information overload (*cf.* Khairat et al., 2018). A dashboard (or cockpit) presents essential information for a defined subject area in an intuitive and understandable way. Dashboards should be designed so that users can get an overview of the information and can derive clear recommendations from this. Empirical evidence suggests that dashboards in a medical context have a positive impact on situational awareness and cognitive load. However, there is a lack of evidence that dashboards reduce information overload. In addition, other studies highlight the benefits of well-designed electronic patient records (e.g., Ahmed et al., 2011; Tan et al., 2013).

When implementing such systems in the medical field (as well as in other application areas), attention should be paid to differences in information processing according to work experience (Wu et al., 2016; Ries and Deml, 2019) as well as to the information needs of different task areas (Wnuk et al., 2016). Overall, there are few studies on interventions to prevent information overload at the level of information design. The overarching recommendations for information design can be found in guidelines for display design or more generally in the field of software ergonomics.

A relatively large number of measures to reduce information overload relate to the personal level. These measures include recommendations on how to manage e-mails or the Internet, specific training as a personnel development measure, and coping strategies to mitigate the negative effects of experienced information overload. Training approaches may include the use of software solutions and the development of more general competencies, such as digital literacy, information literacy, media and information competence, and self and time management (Drössler et al., 2018). In general, the recommendations at the individual level overlap with literacy as a technostress inhibitor (Jena, 2015). Descriptions of concrete training concepts can be found in Moser et al. (2002) and in a program of the “New Quality of Work” initiative (Bundesanstalt für Arbeitsschutz und Arbeitsmedizin (BAuA), 2006). However, there is a lack of methodologically sound empirical studies to evaluate the effectiveness of such training approaches. Recommendations are largely derived from qualitative interview studies, and evaluation studies to date have not assessed actual reductions in information overload. Regarding the use of e-mail and the Internet, recommendations include negotiating and formulating clear organizational rules. In addition, there are several recommendations for the individual handling of e-mails. Other approaches at the personal level include the development of an active boundary between work and leisure time and the improvement of time-and self-management.

At the task and process level, Piecha and Hacker (2020) developed numerous recommendations based on a multi-method research project. In the area of task and process design, however, the empirical effectiveness of the recommendations for action presented has not been comprehensively evaluated. An exception to this is the evaluation of an online intervention for structuring teamwork (STROTA; Ellwart et al., 2015), which reported a reduction in information overload in the intervention group compared to the control group. In general, promoting situational awareness seems to be a recommendable mechanism for managing complex information. Especially in complex decision-making situations, filtering and prioritizing information may be relevant (Bergström et al., 2010). Constructive and supportive leadership behavior is generally discussed as a resource for coping with work-related stress. However, we could not identify concrete intervention approaches and training programs for managers.

Approaches at the organizational level are probably the least separable from the other levels of intervention, since the introduction of new software solutions, the development of human resources, and the design of tasks and processes should also be embedded in the overall strategy of an organization. Therefore, some of the recommendations collected at this level refer to training approaches (e.g., Moser et al., 2002). In addition, the provision of adequate technical solutions, company agreements for the management of digital communication, IT security, and technical support are also addressed in the literature. Together with the techno-inhibitors of participation and support for innovation (Jena, 2015), these factors provide some guidance on how to reduce the potentially harmful effects of information overload and technostress.

On the level of information technology, general recommendations can be found for the selection of suitable communication media depending on the task (e.g., Kauffeld et al., 2016). Furthermore, algorithm-based approaches for filtering as well as extracting and summarizing information are presented in the literature. A unique intervention method is the automation of monitoring tasks. Overall, many studies in this area present basic recommendations as well as algorithmic concepts. At this level, field studies that quantify the effects of interventions on information overload using an experimental control group design are still largely lacking.

The implementation of structural preventive measures represents a process of change within a company, and this implementation requires good planning and, if possible, participatory design and implementation (Rigotti et al., 2014). Ideally, according to Eppler and Mengis (2004), all levels of interventions mentioned in this review should be considered together when planning measures. Indeed, the redesign of the organizational information flow, possibly accompanied by technical assistance systems (e.g., software solutions), also requires the development of personal competencies. In our view, the recommendations for action presented here to reduce perceived information overload, which have been separated according to intervention levels, should not be considered in isolation but should be considered together and coordinated when planning and implementing appropriate measures.

Particularly in the area of information design and the development of information technology approaches, it is evident that the interventions to prevent information overload must be adapted to the concrete work requirements of the respective sector or occupational field. Therefore, no solution is equally suitable for all areas of application without adaptation, and the development of measures should be preceded by an analysis of the current focal points of stress in that area.

The contribution of this literature review is threefold. First, previous general review articles on information overload focused on psychological factors that influence the perception of information overload (Eppler and Mengis, 2004; Antoni and Ellwart, 2017). However, Antoni and Ellwart (2017) did not include technical measures to reduce information overload at all, while the article by Eppler and Mengis (2004) includes these aspects but is almost two decades old, highlighting the importance of an update. Second, a more recent meta-analysis shows the current state of research but is very narrow in its scope (Graf and Antoni, 2020). It focuses on the characteristics of information. We go beyond this meta-analysis by covering the five levels of causes of information overload. As our review shows, the information itself cannot be considered in isolation, since it is received and processed in the social context of tasks, team processes, and the organizational rules. Third, other review articles come from specific occupational fields and therefore only present countermeasures against information overload that are specific to the tasks of this occupation (e.g., Khairat et al., 2018). Although these countermeasures are partially generalizable to other occupations, these reviews omit important findings from other fields.

### 5.2. Strengths and limitations

The temporal limitation of this review to studies from the years 2000 to 2021 is justified by the rapid changes in digital information technologies, as the applicability of older studies to current work processes would be limited. Despite a systematic literature search following the recommendations of the PRISMA standards (Moher et al., 2009), it cannot be ruled out that relevant publications were not identified. In particular, general design knowledge, for example, from the field of software ergonomics, is not a comprehensive part of the present report due to the search strategy applied. Similarly, it may be possible to derive further design recommendations from a systematic review of studies that examined cause-effect relationships (predictors, consequences, mediators, and moderators) for the phenomenon of information overload. The findings summarized in this report should be understood as a systematic presentation of the applied combination and linkage of key terms. By using five of the most important databases for scientific publications (Web of Science, Ebscohost, Medline, PsycInfo, and PsycArticles), good coverage of the relevant literature can be assumed. We supplemented the search in the scientific databases by searches in PSYNDEX Interventions, Rehadat, and Arbeitssicherheit.de and a review of the publications of the BAuA (Federal Institute for Occupational Safety and Health) and the VBG.

All identified publications were assessed in a blinded manner by two independent raters against the defined inclusion and exclusion criteria. Conflicting assessments of specific studies were discussed by the project team. Due to the large heterogeneity of the publications and study formats, a formal assessment of the quality of the evidence base was not performed. However, references to the validity and generalizability of individual findings and recommendations for action were included in the text. Restricting this review to randomized controlled rials in the field of intervention evaluation, as suggested for the COCHRANE reviews, would not have been feasible. Overall, after reviewing and synthesizing the available studies, there is still a considerable lack of robust empirical evidence on the effectiveness of specific interventions to address information overload. Many studies are based on intuitive experience, do not allow for causal inference, or have other methodological limitations.

As a structuring framework, this review used the classification according to Eppler and Mengis (2004), which distinguishes between aspects of information, the person, tasks and processes, organizational processes, and information and communication technology. However, it was not always possible to assign interventions clearly to one of these levels. Here, we have decided to make an assignment in each intervention case, but in the sense of a socio-technical approach, an integration of measures from the different levels is considered necessary.

### 5.3. Conclusion

The studies included in this review cover a wide range of possible approaches to preventing or improving information overload. In terms of concrete information is concerned, it is important to clarify what information is actually relevant and to present this information in a clear and adaptive manner. The studies in this review present a variety of methods for managing large amounts of information for individual employees, some of which have been translated into concrete interventions. Clarifying and structuring team collaboration can also prevent information overload, and managers have a special role to play in this context. Decision-makers at the organizational level are responsible for selecting appropriate software, as well as for transparency and internal company rules that clarify information management. Technological support can help to reduce the amount of information present by using filtering systems or tools to extract the relevant information.

It is striking that the intervention studies in the literature evaluating specific tools or training programs have rarely used a true control group design. Furthermore, it should be noted that information overload has rarely been considered as an outcome. This is particular true at the level of information and information technology. In many studies, the performance or the subjective satisfaction with a tool was often measured instead. A relatively high number of studies used qualitative methods to investigate the need for intervention or existing strategies to deal with information overload. Regarding the use of interviews with employees and asking questions about the strategies they use, it should be noted that this methodology helps to identify what individuals are already doing; therefore, it is often unclear how successful these strategies are and whether they are appropriate starting points for interventions.

Although the individual level has been the most studied in terms of the number of studies, the number of findings related to systemic prevention of information overload has been significantly higher than for behavioral prevention approaches. Thus, the literature of the past 20 years on information overload has already considered the effects of external factors on information overload, such as task characteristics, processes, and technical support, in addition to training and coping measures at the individual level (Meyer et al., 2021).

## Author contributions

All authors listed have made a substantial, direct, and intellectual contribution to the work and approved it for publication.

## Funding

This review is an edited and shortened version of an unpublished report delivered to the Bundesanstalt für Arbeitsschutz und Arbeitsmedizin [Federal Institute for Occupational Safety and Health], which provided funding.

## Conflict of interest

The authors declare that the research was conducted in the absence of any commercial or financial relationships that could be construed as a potential conflict of interest.

## Publisher’s note

All claims expressed in this article are solely those of the authors and do not necessarily represent those of their affiliated organizations, or those of the publisher, the editors and the reviewers. Any product that may be evaluated in this article, or claim that may be made by its manufacturer, is not guaranteed or endorsed by the publisher.
